# Extra-dural epidermoid cyst of the parasellar region: A rare case report

**DOI:** 10.1016/j.ijscr.2024.110322

**Published:** 2024-09-19

**Authors:** Dewa Putu Wisnu Wardhana, Robert Euro Djojoseputro

**Affiliations:** a Neurosurgery Division, Department of Surgery, Faculty of Medicine, Universitas Udayana, Academic Hospital of Universitas Udayana, Badung, Bali, Indonesia; b Resident Medical Officer, Siloam Hospitals Bali, Badung, Bali, Indonesia

**Keywords:** Intracranial epidermoid cyst, Parasellar tumor, Pterional approach

## Abstract

**Introduction:**

Intracranial epidermoid cysts (IECs) comprise less than 1 % of intracranial tumors. IECs begin forming at birth and slowly grows in size. Cerebellopontine angle is the most common location reported. Brain magnetic resonance imaging (MRI) plays crucial role in diagnosis. Gross total resection is the ideal management but adhesion to adjacent structures is often challenging.

**Case presentation:**

We presented a case of 57-year-old female with vertigo and chronic abnormal sensation in her left side of the face. Brain MRI suggested an IEC located in the left parasellar region, which compressed the left trigeminal nerve. Resection was performed through a pterional approach. Gross total resection was successful without injuring nearby structures. Histopathological examination confirmed the diagnosis of epidermoid cyst. Post-operative care was uneventful and patient was discharged 3 days after surgery.

**Discussion:**

Less commonly located IEC in the parasellar region, instead of the cerebellopontine angle, results in compression of the trigeminal nerve. Surgery is appropriate for symptomatic cases. Surgical resection through a pterional approach provided access to the deeply located lesion in the parasellar region. Gross total resection should always be attempted while considering the risk of injuring nearby structures.

**Conclusion:**

Despite IECs being rare intracranial lesions with frequent adhesion and high rate of recurrence, gross total resection should always be attempted. Pterional approach provided great access for intracranial mass located in the parasellar region. Longer follow-up is suggested to monitor clinical outcome and also recurrence.

## Highlights

• Intracranial epidermoid cysts comprise less than 1 % of all intracranial tumors.
• Extra-dural epidermoid cyst located in the parasellar region is an unusual occurrence.
• Histopathologic examination should be performed to confirm the diagnosis.
• Surgical resection is the principal therapy in symptomatic cases.
• Great clinical improvements were achieved following total gross resection of the lesion.

## 1 Introduction

Intracranial epidermoid cysts are rare congenital lesion lined by keratin wall that account for less than 1 % of intracranial tumors. Formation of epidermoid cysts starts during embryogenesis and grows with age at a slow rate. Cerebellopontine angle is the most common location for intracranial epidermoid cysts [1]. While brain magnetic resonance imaging plays an important role in diagnosis, only histopathological study can confirm the diagnosis. Surgical excision is considered in symptomatic cases. Gross total resection minimizes the rate of recurrence, but frequent adhesion to adjacent structures restricts the surgeon's attempt at total removal. Long term follow-up should be performed to anticipate recurrence [2]. We present a case of intracranial epidermoid cyst in the parasellar region where a total gross resection was achieved. This article was written according to the SCARE guideline for case report [3].

## 2 Case report

A 57-year-old female presented to neurosurgery outpatient clinic complaining of dizziness and spinning sensation that started 2 weeks prior to admission. The symptoms were intermittent initially, but lately it was getting more frequent and intense. The patient also mentioned abnormal sensation on the left side of her face. It was described as a numbness that had appeared in the past year. Any painful sensation, impaired taste, or any history of trauma was denied. No significant findings were found on clinical examination aside from impaired sensation in left side of her face. Motoric muscle functions of her face were still intact bilaterally.

**Fig. 1:**
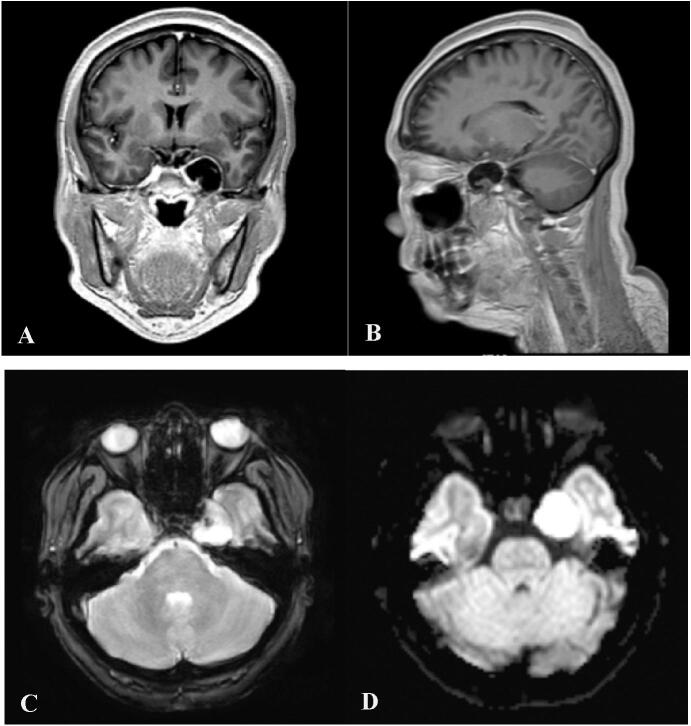
Brain MRI Images reveal an extra-axial mass in the left parasellar region. (A) T1-weighted image showing hypointense signal, (B) Sagittal view of the parasellar bones does not show any signs of infiltration from the mass (B) T2-weighted images showing hyperintense signal that is isointense to CSF, (C) DWI image showing hyperintense signal.

Magnetic Resonance Imaging (MRI) with contrast of the brain (Fig. 1) was performed, revealing an extra-axial mass lesion in the left parasellar region. On T1-weighted images, the mass showed a hypointense signal, while T2-weighted images revealed hyperintensity that was isointense to cerebrospinal fluid. Diffusion-weighted imaging (DWI) showed an enhancement around the periphery of the lesion. The lesion was measured at 2,76 cm × 2,27 cm × 2,44 cm. Medially, the lesion just abutted the cavernous segment of the left internal carotid artery and the adjacent left trigeminal nerve.

**Fig. 2:**
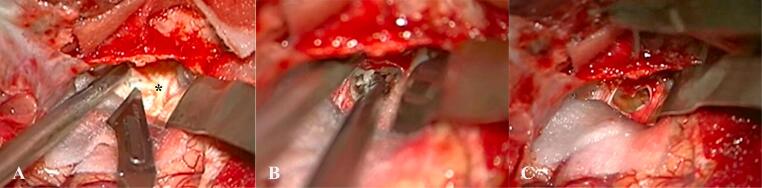
Intraoperative images viewing over the temporobasal region. (A) The dura layer (asterisk) of the temporobasal region before the incision. (B) The content of the cyst, white pearly material with liquid. (C) The successful attempt for gross total resection (B).

Based on clinical history and findings on examinations, the patient was offered a surgical management to resect the mass. The neurosurgeon performed a pterional approach to access the tumor. The duramater in temporobasal region was initially explored and the tumor was identified. Intraoperative findings confirmed the extra-dura nature of the tumor. The dura overlying the mass was hardened and relatively stiff. Incision was made and the content of the tumor was extracted. It contained pearly white substance with yellowish fluid (Fig. 2). The trigeminal nerve compression was relieved after the resection without any significant injury to major arteries. The space was packed with hemostat and then the dura was sutured. A sample of the specimen was sent for histopathological studies. Total removal of the mass was accomplished. Microscopic examination of the specimen revealed basket weave keratin and compact orthokeratosis. Post-operative course was uneventful and the patient was discharged 3 days after the surgery with observed clinical improvement.

**Fig. 3:**
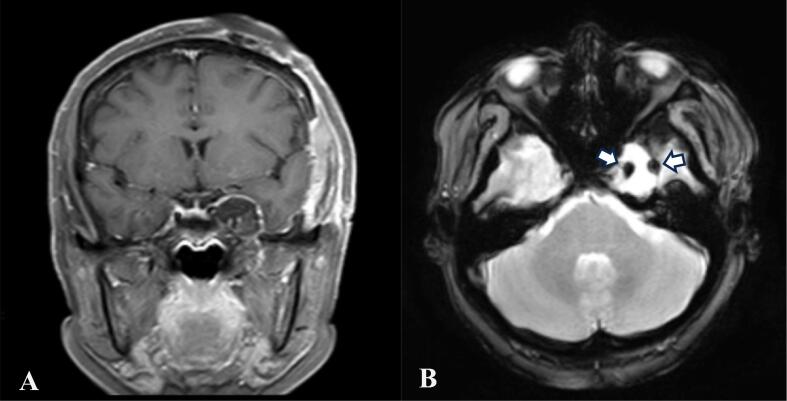
Post-operative images at 2 months follow up, revealing no signs of delayed post-operative bleeding nor tumor recurrence. (A) The dura of the left temporobasal region retaining its shape, hemostats are still observed filling the space. (B) Marks (white arrows) from the incision and sutures are seen in the T2 images.

Follow up at one month post-surgery, the patient presented without abnormal sensation in the face nor vertigo. At 2 months follow-up, patient reported no new neurological deficits. The patient had a repeat brain MRI (Fig. 3) which revealed no signs of recurrence or delayed post-operative bleeding. Hemostats were still observed occupying the space with no compression of the surrounding structures.

## 3 Discussion

Intracranial epidermoid cyst (IEC) is a rare congenital malformation that developed during the 3rd to 5th week of embryonic gestation. This condition arises from the epithelial tissue remnants of the early stages during the fetal developmental stages. IECs account for less than 1 % of all intracranial masses [4]. Most IECs were found in the cerebellopontine angle but previous reports presented IECs residing in the ventricles, brainstem, temporal lobe, and even other parts of the brain [5,6]. Rate of growth is relatively slow with IEC which produces minimal signs and symptoms in the early phases. However, IEC has a great tendency to adhere to the surrounding tissue, in this case the neurovascular structures. Compression by IEC results in neurologic impairments according to its location. Though considered as a benign tumor, IEC has the potential to cause severe morbidities if left untreated [1].

Presenting signs and symptoms are variable depending on the location and the compression to the nearby structure. Most common neurological manifestations are headaches, dizziness, facial nerve palsy, hearing loss, cerebellar signs, and even seizures. Non communicating hydrocephalus might occur in IEC growing in the ventricles [1,7]. In this case report, the patient presented with worsening vertigo and a long history of hemifacial numbness. These symptoms are less commonly reported and thought to be attributed to its uncommon location.

Intracranial epidermoid cysts are best evaluated with brain magnetic resonance imaging (MRI). IECs can be seen as a hypodense lesion on computed tomography images which can be hard to be distinguished from other intracranial lesions. However, MRI demonstrate IECs as hypointensity on T1-weighted images, hyperdensity which is almost isointense to cerebrospinal fluid on T2-weighted images, and distinctive diffusion restriction on diffusion weighted imaging (DWI) [8]. IECs can be distinguished from dermoid cysts by the off midline location and hyperintensity seen on T1-images. Arachnoid cysts appear similar on T1 and T2 images, however DWI will not reveal a hyperintense signal [9]. Our findings in this case exhibited similar radiographic features to the literatures we found. As for the location, we found the lesion to be an extra-dural mass situated in the left parasellar region. The left trigeminal nerve was also compressed by the tumor at the Meckel's cave entrance. No invasion or destruction of the parasagittal bones were noted in our case.

Surgical excision with gross total resection is considered the principal management for IECs. The most frequent challenge confronted is the adhesion to the surrounding neurovascular structures. Retrosigmoid approach plays an important role in epidermoid cysts in the cerebellopontine angle, but different approaches had been previously reported for lesions located in the chiasma, suprasellar region, and the carotid cisterns [10]. However, gross total resection is reported to be achieved in only 50–80 % of IECs [11]. Subtotal resection should be considered in order to avoid unnecessary injuries to the nerves or vascular structures. Recent meta-analysis study estimated the overall recurrence for IECs in the range of 11 % for all procedures. Subtotal resection yielded a higher rate of recurrence, namely 7 times higher, compared to gross total resection [12]. Pterional approach was chosen in our case due to the parasellar location. The tumor was observed to be containing pearly white material with little amount of yellow fluid. Despite the presence of adhesion, gross total resection of the tumor was achieved without injuring nearby neurovascular structures.

## 4 Conclusion

Despite IECs being rare intracranial lesions with frequent adhesion and high rate of recurrence, gross total resection should always be attempted. Pterional approach provided great access for intracranial mass located in the parasellar region. Longer follow-up is suggested to monitor clinical outcome and also recurrence.

## Provenance and peer review

Not commissioned, externally peer-reviewed.

## Consent

Written informed consent was obtained from the patient for publication and any accompanying images. A copy of the written consent is available for review by the Editor-in-Chief of this journal on request.

## Ethical approval

The hospital ethical committee considered an ethical approval is unnecessary since this case report involves no experimentation on humans or animals, rather a rare case encountered during clinical practice.

## Funding

N/A.

## Author contribution

Both authors have contributed equally in the writing of this case report.

## Guarantor

All of the authors should be considered as the guarantors.

## Research registration number

N/A.

## Declaration of generative AI and AI-assisted technologies in the writing process

Preparations of this work didn't involve any AI and/or AI-assisted technologies.

## Conflict of interest statement

The authors report no declarations of interest.
